# Boundarylessness and sleep quality among virtual team members – a pilot study from Germany

**DOI:** 10.1186/s12995-020-00281-0

**Published:** 2020-10-07

**Authors:** Elisabeth Rohwer, Ann-Christin Kordsmeyer, Volker Harth, Stefanie Mache

**Affiliations:** Institute for Occupational and Maritime Medicine (ZfAM), University Medical Center Hamburg-Eppendorf (UKE), Seewartenstraße 10, Haus 1, 20459 Hamburg, Germany

**Keywords:** Digitalisation, ICT, Psychological detachment, Perceived stress, New work, Virtuality, Virtual job demands, Virtual teamwork, Health promotion

## Abstract

**Background:**

In the course of globalisation and digitalisation, new ways of work are becoming increasingly prevalent. To remain competitive as an organisation, cooperation across time, place, and organisational boundaries is becoming necessary. Virtual teamwork offers these advantages, but can also be both, an opportunity and a burden, for employees. This pilot study aims to gain first insights into job demands and resources in virtual teamwork to provide a basis for further research from which appropriate health promotion and prevention measures can be derived.

**Methods:**

In this pilot study, an online questionnaire was used to examine the relationship between boundarylessness as a job demand, psychological detachment as a personal resource, as well as perceived stress and sleep quality as health outcomes among 46 virtual team members from Germany. Data collection lasted from October 2019 to January 2020. Validated scales were used for the questionnaire, except for virtuality. Due to insufficient operationalisation to date, a virtuality scale was developed based on the current state of research. The data were analysed with ordinal logistic regression analyses and median split *t*-tests.

**Results:**

The results indicate that perceived stress impaired sleep quality of virtual team members in this sample. In contrast, successful psychological detachment from work was positively related to sleep quality. A higher degree of virtuality coincided with higher levels of boundarylessness. Virtual team members with leadership responsibility showed higher levels of psychological detachment.

**Conclusion:**

The present pilot study breaks ground and provides initial insights into the relationship between virtual teamwork and employee health in the German context. Further research, particularly on job demands in virtual teamwork, is needed to derive concrete health promotion and prevention measures.

## Background

### Virtual teamwork as a new way of work

With the advent of new communication technologies, new forms of collaboration are becoming increasingly prevalent. To address increasing globalisation and digitalisation, organisations implement project-based virtual teams that allow them to act very flexibly on a volatile market [1]. In a worldwide survey, 89% of 1620 respondents from 90 countries reported working on a virtual team [2]. Virtual teams consist of members who collaborate across distances of space and time [3]. In contrast to “traditional” teams, virtual team members are distributed among different locations, collaborating interactively based on a common task and/or goal [3, 4]. They are thus interdependent and share responsibility for outcomes [5]. Their communication is based on information and communication technologies (ICT), such as emails or video calls [3]. The transition from “traditional” to virtual teamwork is considered to be gradual rather than dichotomous i.e., teams may adapt higher or lower degrees of virtuality [3, 5]. They might also assume a hybrid shape, working face-to-face from time to time as well as never meeting each other in person [3, 6]. Examples of virtual teams range from team members in different departments or cities to intercultural, transnational or globally dispersed teams with members from different companies [4, 7].

Parallel to these trends, another development is emerging that primarily affects employees, but also organisations, the national economy and community: since 2008 the number of mental disorders among employees has been increasing by 64.2% and, along with musculoskeletal disorders, has become the main cause of absenteeism and unemployability [8]. The cost of mental disorders alone amounts to 44.4 billion euros per year in Germany [9]. This development includes virtual team members. Previous studies on virtual teamwork mainly focussed on challenges and job demands, assuming that working conditions in virtual teams are more stressful than in collocated teams [10]. Despite the growing media and research attention for occupational health, the adaptation of mental health issues in the context of virtual teamwork has been scarce in research up to now [11]. Although a few researchers have addressed both, challenges and job-related health outcomes in virtual teamwork [11–14], a dearth of research remains.

### Study aims

To fill this void, this study aimed to gain first insights into job demands and resources as well as resulting health outcomes in virtual teamwork. These initial insights may provide a starting point for further research and the deduction of adequate health promotion and prevention measures in the context of virtual teamwork. The rapidly increasing implementation of virtual teams worldwide highlights the relevance of this study.

### Theoretical background

We based this study on the theoretical framework of the Job Demands-Resources Model (JD-R Model) [15]. This model was chosen because it extended the ideas of the job demand-control model [16] and the effort-reward imbalance model [17], and includes both demanding and beneficial aspects and processes of employees’ working environments [15]. One main advantage of the JD-R Model lies in its adaptability and applicability to numerous occupational settings [18], as a myriad of empirical studies based on this theoretical framework show [19]. The JD-R Model considers aspects of the job that require sustained effort associated with physiological or psychological costs, potentially triggering a health-impairing process that predicts psychosomatic health complaints. However, the model also considers resources that lie in the job itself or the person performing it. While job demands are negatively associated with health outcomes, job and personal resources are positively related to them and may help employees to cope with job demands [19]. A high level of resources may also lead to higher motivation and keep strain reactions on a medium level. Moreover, resources can serve as a buffer for job demands, reducing psychological costs and stimulating personal growth, learning, and development [20, 21]. To reach our objective of gaining first insights into virtual team members’ mental health, we examined boundarylessness as a job demand in virtual teamwork and investigated psychological detachment as a personal resource to cope with this demand. Perceived stress and sleep quality were assessed as health outcome variables. Additionally, perceived stress was examined as a mediator between boundarylessness and sleep quality.

### Boundarylessness in virtual teamwork

Although virtual teamwork promises many advantages, such as high flexibility, virtual team members face particular challenges due to their working conditions [11–14]. Not only do they depend on ICT and lack regular face-to-face contact, working across geographical distances and time zones can also lead to expectations and practice of permanent availability among virtual team members [6]. Consequently, virtual team members have decreased possibilities to manage their work-life boundaries, personal time and phases of recovery [22]. This boundarylessness refers to both, time and space [23]. Facilitated by digitalisation, this can easily lead to an intrusion of working life into private spheres [23, 24]. There is evidence that permanent availability, as one aspect of boundarylessness and frequent ICT usage, creates stress among employees [14, 25]. Scientific literature refers to the inability to cope with new technologies as “digital stress” which is associated with health impairments and sleep disturbances [26]. Sleeping troubles are not only associated with perceived stress but also with characteristics of our globalised, highly digital and flexible “24-h society” [27]. These particular working conditions make virtual team members especially susceptible to experience boundarylessness [28]. Based on these findings, we formulate the following hypotheses:

- ***Hypothesis 1****: There is a significant negative relationship between boundarylessness and sleep quality.*
- ***Hypothesis 2****: There is a significant positive relationship between boundarylessness and perceived stress (H*_*2a*_*) and a significant negative relationship between perceived stress and sleep quality (H*_*2b*_*). Perceived stress partially mediates the relation between boundarylessness and sleep quality (H*_*2c*_*).*

Proceeding from recent findings, we assume that virtuality rather poses a challenge to team members [10]. There is first evidence from qualitative research that virtual teamwork is associated with stress [13]. Consequences of stress range from somatic and somatoform to mental disorders [29]. The association of sleep disturbances with digital stress and flexible working hours, as well as their high prevalence of up to 42% among German employees [26, 30], lead to the third hypothesis:

- ***Hypothesis 3****: There is a significant positive relationship between virtuality and perceived stress (H*_*3a*_*). The degree of virtuality moderates the relation between boundarylessness and perceived stress in such way that a higher degree of virtuality amplifies the positive relation between boundarylessness and perceived stress (H*_*3b*_*). The degree of virtuality moderates the relation between boundarylessness and sleep quality in such way that a higher degree of virtuality amplifies the negative relation between boundarylessness and sleep quality (H*_*3c*_*).*

### Psychological detachment in virtual teamwork

Especially when working “anytime anywhere” [31], mental disengagement from work-related duties becomes an important personal resource [32, 33]. Psychological detachment is one aspect of recovery experiences and describes the act of leaving work not only physically, but rather mentally [34]. A lack of psychological detachment is associated with adverse health outcomes and reduced well-being [35], such as perceived stress [36] and diminished sleep quality [37]. Recent findings highlight the relevance of psychological detachment as a personal resource to cope with boundaryless work and sleeping problems [38]. Therefore, we assume that psychological detachment can be an important resource and coping mechanism for virtual team members. Based on these findings, we formulate the following hypothesis:

- ***Hypothesis 4****: There is a significant negative relationship between psychological detachment and perceived stress (H*_*4a*_*). The ability to detach from work moderates the relation between boundarylessness and perceived stress in such way that psychological detachment attenuates the relation between boundarylessness and perceived stress (H*_*4b*_*). The ability to detach from work moderates the relation between boundarylessness and sleep quality in such way that psychological detachment attenuates the relation between boundarylessness and sleep quality (H*_*4c*_*).*

**Fig. 1:**
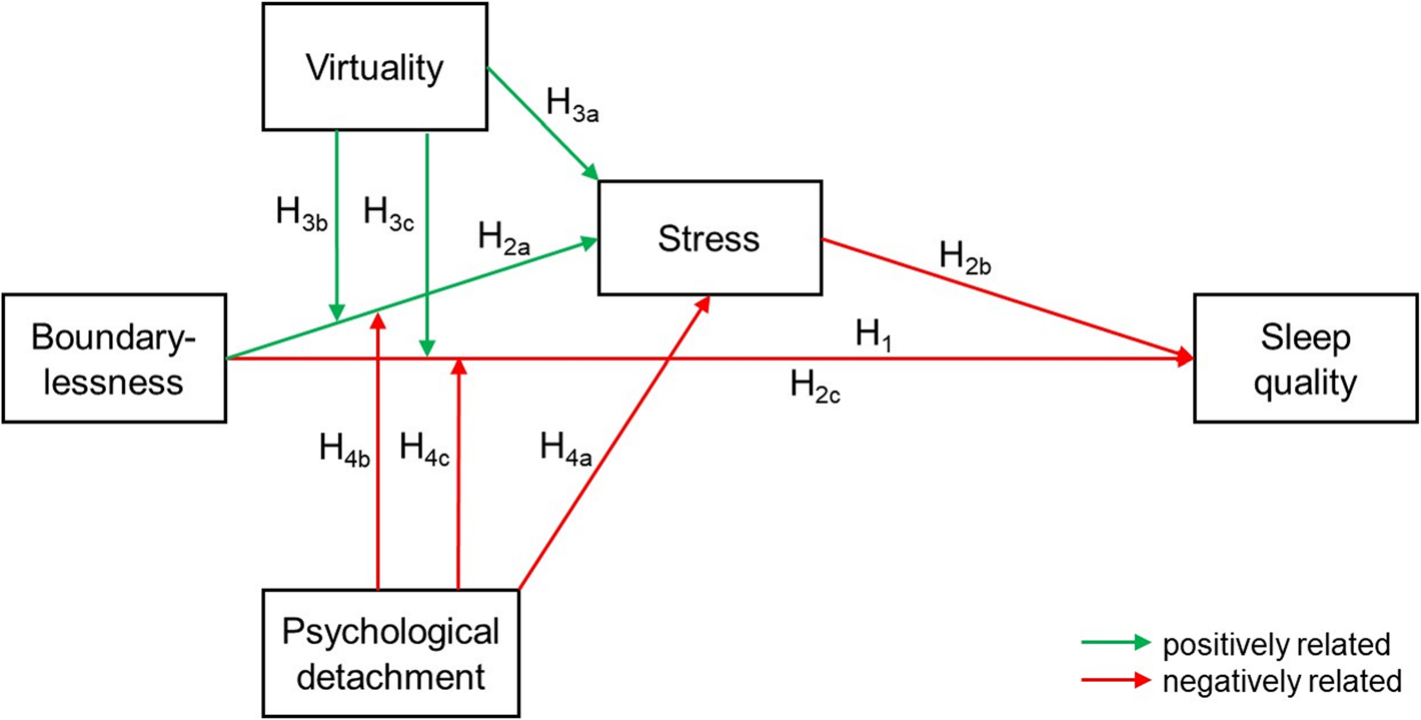
Conceptual model of formulated hypotheses

A conceptual model of all formulated hypotheses is provided in Fig. 1.

## Methods

### Sample and procedure

The study was conducted as a cross-sectional pilot study, using an online questionnaire. Participants were recruited and the data were collected between October 2019 and January 2020. The sample was generated incidentally, without stratification or random selection. No grouping, such as a control group, was performed. Links to the online questionnaire were sent to the contacted organisations and then internally forwarded to virtual team members by email. As the questionnaire was written in German and participants for the pilot study were recruited in Germany, sufficient German language proficiency was required for participation. Participants were included in data analyses based on the following inclusion criteria, (1) who worked as employees (excluding freelancers or self-employed workers), (2) who had work experience in their current profession of at least 1 year, (3) who worked full time (at least 35 h per week), and (4) who worked in virtual teams. Virtual teamwork was operationalised focussing on:

- the frequency of using different communication technologies, such as text messages, phone or video calls
- the frequency of face-to-face meetings among virtual team members
- the number of virtual team members that work at the same site
- the number of sites over which virtual team members are distributed
- the geographical distance between virtual team members

Due to the smaller scope of this national pilot study, factors such as time asynchronicity and differences in language and/or culture were assessed additionally and evaluated descriptively, but not included into the operationalisation of virtuality. Participants were recruited based on a list of IT companies provided by the Hamburg chamber of commerce and research of organisations in Germany deploying virtual teams i.e., predominantly medium-sized and large companies, via professional social networks, mailing lists, and online communities.

### Data measurement

Based on the JD-R Model [15] and our hypotheses, boundarylessness was assessed as a job demand and independent variable. Virtuality and psychological detachment were examined as independent variables and moderators on the relationship between boundarylessness and the two outcome variables perceived stress and sleep quality. Perceived stress was also investigated as a mediator between boundarylessness and sleep quality, as shown in Fig. 1. The data were collected based on self-reports, using an online questionnaire. All surveys contained anonymous data only. Table 1 provides an overview of the main variables and their measurement.

**Table 1:** List of main variables and measurement

Construct (type of variable)	Measurement and source	Number of items
Boundarylessness (IV)	Boundarylessness subscale of Work 4.0 questionnaire [39]	5
Virtuality (IV, moderator)	Self-developed scale based on previous operationalisations [40–43]	8
Psychological detachment (IV, moderator)	Psychological detachment subscale of Recovery Experience Questionnaire [34]	4
Perceived stress (IV, mediator, DV)	Perceived Stress Scale [44]	10
Sleep quality (DV)	Sleeping troubles subscale of Copenhagen Psychosocial Questionnaire (COPSOQ) [45]	4

*Note. IV* Independent variable, *DV* Dependent Variable

#### Sociodemographic and workplace variables

Self-constructed as well as already established items were used to assess job category [46], industry [47], job title, professional work experience [48], size of the organisation [49], type of employment, leadership responsibility [50], working hours, project work [51], age, gender, nationality, federal state, and education level [47].

#### Job demands

Boundarylessness was assessed using the reliable and validated boundarylessness subscale of the Work 4.0 questionnaire [39]. The scale consists of five items on a five-point Likert scale (1 = *strongly disagree*, 2 = *disagree*, 3 = *neither agree nor disagree*, 4 = *agree*, 5 = *strongly agree*). The item “I can be reached by my colleagues and superiors at any time during my vacation.” exemplifies the scale. The authors of the scale reported good values of reliability (Cronbach’s α = .78 / .79) and validity [39].

Due to a lack of appropriate timely and consistent measurement of virtuality, we developed a virtuality scale based on existing operationalisations and the current state of research. The overall scale did not show satisfactory values of reliability (Cronbach’s α = .561). However, when removing the items on digital media usage, internal consistency increased considerably (Cronbach’s α = .731). We identified the following eight items for the measurement of virtuality:

“How often do you use the following communication media to communicate with your virtual team members: email/text messages/chats, social media, phone (conference) calls, video (conference) calls or video chats?” on a scale from 1 = *never*, 2 = *less than monthly*, 3 = *at least monthly*, 4 = *at least weekly*, 5 = *at least daily,* adapted from [43].

“How often do you meet your virtual team members face-to-face?” on a scale from 1 = *never*, 2 = *once a year*, 3 = *twice a year*, 4 = *three times a year*, 5 = *more than three times a year*, adapted from [42].

“How many of your virtual team members work at the same site as you do?” on a scale from 1 = *none, I am the only team member at my site*, 2 = *approximately one-quarter of my virtual team members*, 3 = *approximately half of my virtual team members*, 4 = *more than half of my virtual team members*, adapted from [41].

“Over how many different sites are you and your virtual team members distributed?” on a scale from 1 = *we all work at the same site*, 2 = *two different sites*, 3 = *three different sites*, 4 = *four different sites*, 5 = *five or more different sites*, also adapted from [41].

“How far does the majority of your virtual team members work away from you?” on a scale from 1 = *in the same building*, 2 = *in the same city*, 3 = *in another city in the same country*, 4 = *in a different country*, 5 = *on a different continent*, adapted from [40].

An additional “virtual job demands scale” of 13 items was developed to examine specific job demands of virtual team members based on previous research [11]. These items were not included in the operationalisation of virtuality or regression analyses but analysed for descriptive purposes only. One exemplary item from this scale is “Due to reduced face-to-face contacts and the use of information and communication technologies, I perceive a higher susceptibility to errors in communication.” All 13 items of this scale were tested using a five-point Likert scale (1 = *to a very limited extent*, 2 = *to a lesser extent*, 3 = *partly*, 4 = *to a large extent*, 5 = *to a vast extent*). Participants were additionally given the opportunity to note free text answers concerning their experience of virtual teamwork-specific job demands.

#### Personal resources

The psychological detachment-subscale of the reliable and well-validated Recovery Experience Questionnaire [34] was used to measure psychological detachment. The scale consists of four items on a five-point Likert scale ranging from (1 = *strongly disagree* to 5 = *strongly agree*). An example item is “I get a break from the demands of work.” The subscale shows good values of reliability (Cronbach’s α = .89) and is well-validated [34, 52].

#### Health outcomes

We assessed perceived stress using the reliable and well-validated Perceived Stress Scale [44] in its 10-item version. The items are presented on a five-point Likert scale (1 = *never*, 2 = *almost never*, 3 = *sometimes*, 4 = *fairly often*, 5 = *very often*). The original scale ranges from zero to four was adjusted for better comparison to the other scales. An example item is “In the last month, how often have you been upset because of something that happened unexpectedly?” The scale is sufficiently validated and reliable [53].

To measure the overall outcome of sleep quality, the sleeping troubles-subscale of the COPSOQ II was used. The subscale consists of four items presented on a five-point Likert scale (1 = *poor*, 2 = *fair*, 3 = *good*, 4 = *very good*, 5 = *excellent*). One item to exemplify the scale was “How often have you slept bad and restlessly?” referring to the past 1 weeks just like the Perceived Stress Scale. The scale was considered reliable and sufficiently validated [45].

### Statistical analyses

Initially, data were checked for plausibility, revealing no suspicious cases. Single items were recoded, where necessary, and scales were built. Considering the small sample size and partly unmet assumptions for linear regression analysis, we conducted ordinal logistic regression analyses. All assumptions for ordinal logistic regression analysis were met. To prevent cells with zero frequencies within this small sample size, ordinal logistic regressions were run separately for each hypothesis. Furthermore, the five categories resulting from the Likert scales were reduced to three categories based on percentiles to ensure a sufficient size within the categories. Despite the risk of bias, this procedure was considered most adequate. For mediation analysis with multicategorical variables, Hayes’s PROCESS macro version 3.5 for SPSS (model 4) was used [54]. For supplementary analyses, descriptive analyses of further job demands, further regression analyses and median split *t*-tests were conducted. All data were analysed using the IBM® SPSS® Statistics (version 26, IBM, Armonk, NY, USA).

## Results

### Descriptive statistics of the sample

After 3 months of recruitment, a sample size of *N* = 62 completed questionnaires was reached. However, we had to exclude 16 participants from data analysis due to unmet inclusion criteria, resulting in a final sample size of *N* = 46. All participants were German citizens. Most of the participants work in the IT industry (*N* = 40 or 86.9%), in large enterprises (*N* = 25 or 54.3%). However, although this result may be biased by the recruitment strategy, this distribution of industries and enterprise sizes confirms previous findings among virtual teams [2]. Further results are provided in Table 2.

**Table 2:** Sociodemographic and Occupational Characteristics of the Sample (*N* = 46)

Variable	*n*	%
Gender		
Female	11	23.9
Male	35	76.1
Age		
18–20 years	1	2.2
21–30 years	10	21.7
31–40 years	18	39.1
41–50 years	7	15.2
51–60 years	9	19.6
61–70 years	1	2.2
Highest educational level		
Secondary education	9	19.6
Higher education	37	80.4
Leadership responsibility		
Yes	20	43.5
No	26	56.5
Work experience		
1–2 years	13	28.3
3–5 years	16	43.8
6–10 years	7	15.2
≥ 11 years	10	21.7
Average working hours per week		
35–40 h	18	39.1
41–45 h	18	39.1
≥ 46 h	10	21.7

### Descriptive statistics of the main variables

Means, standard deviations, Cronbach’s α, and zero-order correlations of the main variables are provided in Table 3.

**Table 3:** Means, Standard Deviations, Internal Consistencies, and Zero-Order Correlations

Variable	*M*	*SD*	Range	Min	Max	1	2	3	4	5
1. Boundarylessness	2.870	.966	1–5	1.000	5.000	(.866)				
2. Virtuality	3.541	.524	1–5	2.625	4.875	.351*	(.561^a^ / .731^b^)			
3. Psychological detachment	3.011	.823	1–5	1.000	4.500	−.395**	−.215	(.844)		
4. Perceived stress	2.490	.559	1–5	1.700	3.800	.092	.237	−.241	(.843)	
5. Sleep quality	3.766	.706	1–5	2.000	5.000	.018	−.279	.282	−.367*	(.815)

*Note. N* = 46. One-tailed Spearman Correlation Coefficients were used. * *p* < .05. ** *p* < .01. Cronbach’s alphas are listed in parentheses on the diagonal. ^a^Internal consistency for self-developed virtuality scale. ^b^Internal consistency for virtuality scale after removing items on digital media usage

### Descriptive statistics of virtual job demands and health promotion offers

We also evaluated job demands other than boundarylessness using another self-developed scale as well as free text answers. Six participants reported specific job demands, such as a need for clear and measurable objectives, lack of social exchange, required availability and high discipline, as well as different levels of media skills and technological equipment among virtual team members. Results from the descriptive analysis of the virtual job demands scale indicate that the participants of this study rather collaborated with virtual team members within the same time zone, but speaking different languages. Although social exchange among and integration of virtual team members seemed to pose greater challenges to virtual team members in this sample, results show high levels of satisfaction with communication and collaboration within the virtual teams. More details are provided in Table 4.

**Table 4:** Descriptive Statistics for Virtual Job Demands

Item	*M*	*SD*	Min	Max
Team members in different time zones	1.739	1.255	1	5
Team members with other mother tongues or dialects	2.848	1.414	1	5
Team members with different cultural backgrounds	2.935	1.357	1	5
Higher susceptibility to errors in communication	2.457	1.609	1	5
Higher susceptibility to conflicts among virtual team members	2.391	1.105	1	5
More difficulties due to diverse cultural and linguistic backgrounds	1.935	.952	1	5
More difficulties due to geographical distance	2.196	1.046	1	5
Restricted possibility of social exchange	3.130	1.128	1	5
More difficulties of socially integrating virtual team members	3.087	1.226	1	5
More difficulties in performance assessment	2.500	1.169	1	5
Difficulties to build trust in virtual team members	2.304	1.133	1	5
Satisfaction with communication within virtual team	4.087	.812	2	5
Satisfaction with collaboration within virtual team	4.152	.868	1	5

*Note. N* = 46. All scales are five-point Likert scales, ranging from one to five

Regarding organisational health promotion, 54.3% of all participants reported that their employers provided personnel development or health promotion offers, but only 39.1% of those who were offered this opportunity made use of it. The most frequently mentioned measures include teambuilding activities (45.7%), soft skill training (39.1%), and self-management training (37.0%).

### Relationships between boundarylessness, virtuality, psychological detachment, and health outcomes

An ordered logit model was estimated to investigate whether different levels of boundarylessness (“low”, “moderate”, “high”) predict different levels of sleep quality (“poor,” “medium,” “good”), testing hypothesis H_1_. The predictors did not account for a significant amount of variance in the outcome, likelihood ratio χ^2^(2) = .588, *p* = .745. The ordered logit model for hypothesis H_2a_ could not confirm the hypothesised association between boundarylessness and perceived stress (χ^2^(2) = .589, *p* = .745). Hypothesis H_2b_, a negative relation between perceived stress and sleep quality, could be confirmed. The tested ordered logit model indicated that different levels of perceived stress (“low”, “moderate”, “high”) accounted for a significant amount of variance in sleep quality, likelihood ratio χ^2^(2) = 7.667, *p* = .022. Low levels of perceived stress, *b* = 1.747, *SE* = .726, *OR* = 5.737, *p* = .016, and moderate levels of perceived stress, *b* = 1.561, *SE* = .708, *OR* = 4.764, *p* = .027, predicted better sleep quality. Good model fit and a satisfied assumption of proportional odds could be confirmed (χ^2^(2) = 1.284, *p* = .526). These results indicate that for virtual team members of this sample who perceived low or medium levels of stress, the odds of being more likely to enjoy good sleep quality were more than 4–5 times higher than those who perceived higher levels of stress. To test hypothesis H_2c,_ a mediation analysis based on model 4 was conducted using Hayes’s PROCESS macro for SPSS. Following hypotheses H_1_-H_2b_ already tested, of which two could not be confirmed, the assumed mediation effect of perceived stress on the relation between boundarylessness and sleep quality could not be confirmed either (with indirect effects *ab*_*1*_ = .0130, *SE* = .1128, 95% CI [−.2062, .2542] and *ab*_*2*_ = −.0635, *SE* = .1216, 95% CI [−.3619, .1391]). Hypothesis H_3a_ had to be rejected due to lack of significance (χ^2^(2) = 1.315, *p* = .518), indicating no significant relation between the degree of virtuality and perceived stress. Virtuality was not found to be a significant moderator either, therefore denying H_3b_ (χ^2^(6) = 3.878, *p* = .693) and H_3c_ (χ^2^(6) = 8.572, *p* = .199). The assumed significant relation between psychological detachment and perceived stress (H_4a_) could not be confirmed (χ^2^(2) = 1.351, *p* = .509). Both of the assumed moderation effects of psychological detachment could not be confirmed (χ^2^(6) = 3.127, *p* = .793 for H_4b_ and χ^2^(6) = 12.077, *p* = .060 for H_4c_). However, post hoc analyses revealed a significant association of psychological detachment and sleep quality (χ^2^(2) = 6.099, *p* = .047), indicating a positive relation between these two variables. Only lower psychological detachment (*b* = − 1.615, *SE =* .678, *OR* = .199, *p* = .017) predicted sleep quality. The model’s goodness of fit, as well as the assumption of proportional odds (χ^2^(2) = .630, *p* = .730), could be confirmed. Accordingly, we may assume that poor psychological detachment from work decreased the odds of enjoying good sleep quality among this sample of virtual team members. More details are provided in Tables 5 and 6.

**Table 5:** Ordinal Logistic Regressions of Associations Between Predictors and Sleep Quality

Effect	*OR*	95% CI	
		*LL*	*UL*
Boundarylessness			
Low	1.462	.430	4.968
Medium	.892	.221	3.607
High	Ref.		
Virtuality			
Low	2.147	.583	7.909
Medium	.430	.111	1.670
High	Ref.		
Detachment			
Low	.199	.053	.752
Medium	.634	.163	2.469
High	Ref.		
Perceived Stress			
Low	5.737	1.383	23.831
Medium	4.764	1.190	19.068
High	Ref.		
B x V			
Low	.128	.005	3.445
Medium	.441	.042	4.614
High	Ref.		
B x D			
Low	2.504	.130	48.327
Medium	8.298	.915	75.189
High	Ref.		

*Note. N* 46, *OR* Odds ratio, *CI* Confidence interval, *LL* Lower limit, *UL* Upper limit, *Ref*. reference category, *BxV* Interaction term of boundarylessness and virtuality, *BxD* Interaction term of boundarylessness and psychological detachment

**Table 6:** Ordinal Logistic Regressions of Associations Between Predictors and Perceived Stress

Effect	*OR*	95% CI	
		*LL*	*UL*
Boundarylessness			
Low	.661	.194	2.248
Medium	.631	.155	2.557
High	Ref.		
Virtuality			
Low	.504	.139	1.828
Medium	.556	.147	2.104
High	Ref.		
Detachment			
Low	1.978	.564	6.931
Medium	1.033	.269	3.963
High	Ref.		
B x V			
Low	1.931	.082	48.183
Medium	.582	.061	5.607
High	Ref.		
B x D			
Low	1.654	.111	24.656
Medium	.666	.095	4.660
High	Ref.		

*Note. N* 46, *OR* Odds ratio, *CI* Confidence interval, *LL* Lower limit, *UL* Upper limit, *Ref.* Reference category, *BxV* Interaction term of boundarylessness and virtuality, *BxD* Interaction term of boundarylessness and psychological detachment

### Differences in degrees of virtuality

In addition, we conducted median split *t*-tests to examine differences between groups among the sample. Splitting the data into higher and lower degrees of virtuality revealed that those 50% of participants who reported a higher degree of virtuality in their teamwork also reported significantly higher values (*t* = − 2.327, *p* = .025, *d* = .685) of boundarylessness (*M* = 3.200, *SD* = .966, *n* = 22) than those who worked in a team of a lower degree of virtuality (*M* = 2.567, *SD* = .880, *n* = 24).

### Differences in levels of boundarylessness

Moreover, splitting the data into higher and lower levels of perceived boundarylessness resulted in a significant difference with regard to psychological detachment (*t* = 1.138, *p* = .003, *d* = .928). Participants who experienced lower levels of boundarylessness reported higher levels of psychological detachment (*M* = 3.330, *SD* = .742, *n* = 24) compared to participants who experienced higher boundarylessness also reporting lower psychological detachment (*M* = 2.631, *SD* = .764, *n* = 22).

### Differences in levels of psychological detachment

**Fig. 2:**
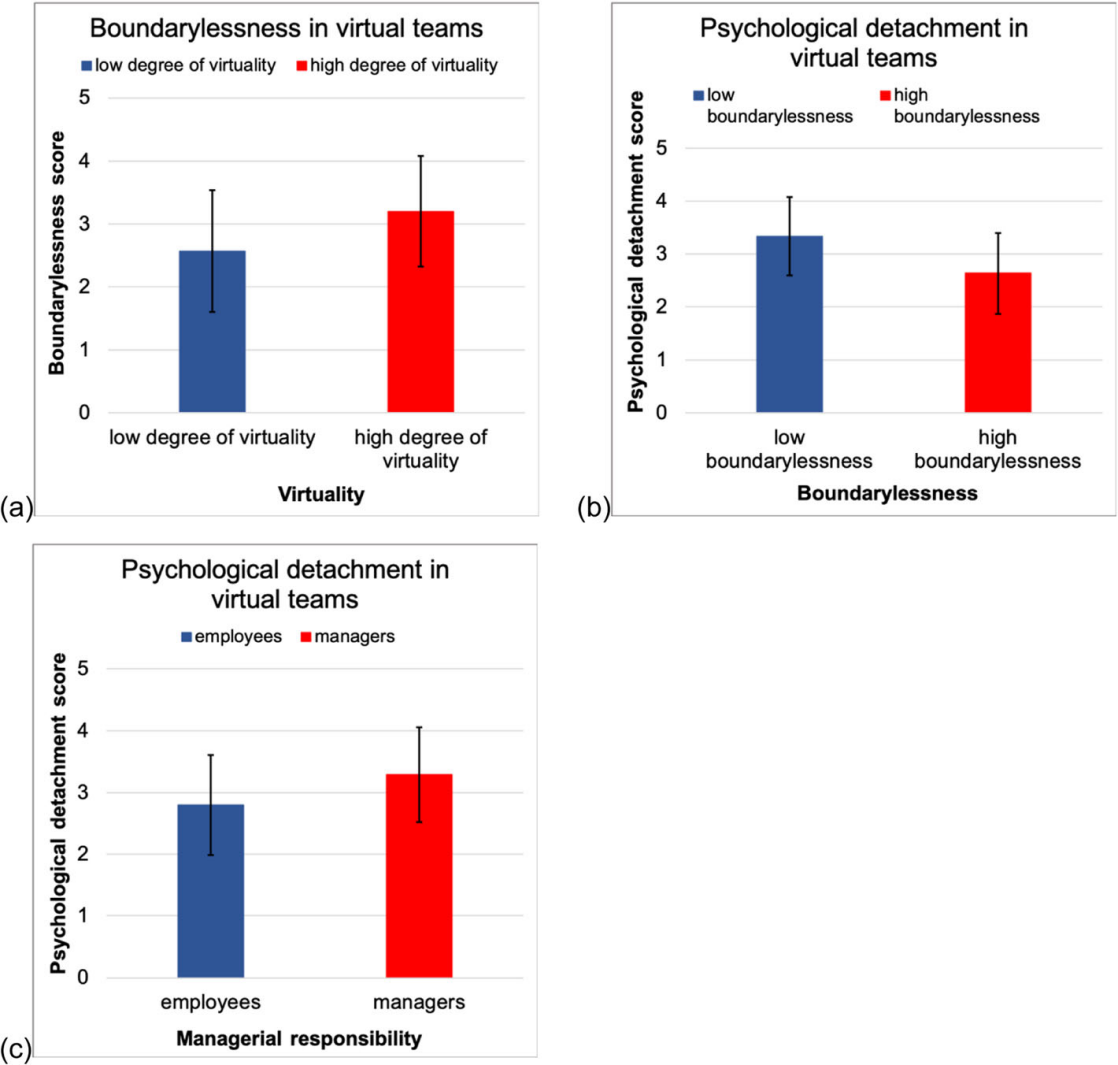
Results from median split t-tests. **a** Differences in degrees of virtuality, **b** differences in levels of boundarylessness, **c** differences among supervisors and employees

We also found significant differences in the levels of psychological detachment between participants with leadership responsibility and those without *t* = 2.071, *p* = .044, *d* = .620). Compared to participants without leadership responsibility (*M* = 2.798, *SD* = .815, *n* = 26), supervisors reported higher levels of psychological detachment (*M* = 3.288, *SD* = .766, *n* = 20). All results from median split *t*-tests are provided in Fig. 2.

## Discussion

The objective of the present study was to examine boundarylessness as a virtual job demand, psychological detachment as a personal resource and perceived stress and sleep quality as related mental health outcomes among virtual team members in Germany. The results revealed significant associations of psychological detachment and perceived stress with sleep quality among virtual team members. The findings indicated potential negative effects of perceived stress among virtual team members as well as beneficial effects of psychological detachment on their sleep quality. Significant differences were found regarding different degrees of virtuality and boundarylessness as well as among supervisors and employees. Yet, the results have to be interpreted with caution due to the small sample size of this pilot study.

### Experience of virtual job demands

Occupational characteristics of the sample show that participants mostly worked in virtual teams within the same time zone. Although they reported some cultural and linguistic differences, they did not seem to perceive them as particularly demanding. Results from our self-developed virtual job demands scale indicate that the restricted possibility of social exchange and difficulties of socially integrating virtual team members concerned participants the most. Optional free text answers support this finding. However, the majority of participants were satisfied with the communication and collaboration within their virtual teams. Interestingly, descriptive data also indicate very long working hours despite a lack of significant relations between boundarylessness, perceived stress and sleep quality. Further research based on this pilot study will be needed to identify resources that explain this finding.

### Relations between boundarylessness, virtuality, psychological detachment and sleep quality

Within the sample of this pilot study, we could not find evidence for a negative relation of boundarylessness or virtuality and self-reported sleep quality. Considering the sample size of this study, further research will be needed to clearly identify boundarylessness as a job demand among virtual team members. The application of longitudinal designs in future research could provide a deeper understanding of these relations.

Results revealed that psychological detachment from work can be a valuable resource for virtual team members as it was positively related to sleep quality. This study therefore transferred already existing evidence of this relationship to the context of virtual teamwork [55, 56]. This finding can be particularly relevant to deduce prevention and health promotion measures in the future. We also found support for a positive relation between perceived stress and sleep quality among virtual team members. Again, this relationship supports evidence from previous research [26, 57] and suggests its applicability to the specific working context of virtual teamwork. Possible adverse health consequences of enduring insufficient sleep highlight the relevance and urgency for further research on health outcomes in virtual teamwork [58, 59]. The fact that only about half of all participants reported that their employer provides health promotion offers emphasises the urgent need to develop appropriate measures for virtual team members to facilitate and support health promotion.

### Relations between boundarylessness, virtuality, psychological detachment and perceived stress

Contrary to our hypotheses, boundarylessness was not related to perceived stress in this study. Reasons for these results may lie in the characteristics of the sample. The descriptive statistics indicate that the majority of virtual team members in this sample did not collaborate across large geographical distances or time zones. Asynchronicity resulting from such collaboration maybe would have amplified experienced boundarylessness, as previous research suggests [41]. Although virtual team members reported perceived stress in a qualitative Finish study, virtuality was not found to be directly related to perceived stress in the present study. One explanation could be a self-selection bias. Possibly, employees only work in virtual teams when they feel attracted to such working conditions or experience high flexibility as beneficial rather than demanding [60]. We did not find support for the predicted mediation of perceived stress. However, the lack of significance of most of the direct paths between the variables explains this finding [61].

### Differences in degrees of virtuality, boundarylessness and psychological detachment

Post hoc median split analyses revealed that participants working in teams with a high degree of virtuality (e.g., long geographic distances between team members) perceived a stronger blending of work and private life whereas participants working in teams with a lower degree of virtuality (e.g., frequent or regular face-to-face meetings with team members) perceived less boundarylessness. This finding supports the assumption that virtual teamwork affects the boundaries between work and private life [28].

Furthermore, participants perceiving high levels of boundarylessness reported lower psychological detachment. These results indicate that virtual team members who experience higher levels of boundarylessness also find it more difficult to disengage from work in their free time. This finding again highlights the urgency of developing prevention and health promotion measures for virtual team members, since higher levels of boundarylessness require even better psychological detachment to deal with work during leisure in a healthy way [32].

Interestingly, higher levels of psychological detachment were found among virtual team members with leadership responsibility. Therefore, it may be assumed that employees have greater difficulty to distance themselves from work during their free time whereas supervisors set a good example by detaching from work more successfully. This result is surprising because it contradicts Latniak’s (2017) finding of higher amounts of burnout among project supervisors compared to employees in virtual contexts [62]. Further research on virtual teamwork should, therefore, examine the role of health-oriented leadership and self-care in virtual teams [63]. However, the results need to be interpreted very carefully regarding this study’s cross-sectional design, limited sample size, and the dichotomisation of the variables for median split analyses [64].

### Strengths and limitations

This pilot study was the first quantitative study to apply an adapted version of the JD-R Model to the context of virtual teamwork in Germany. Our results provide first insights and contribute to obtaining a better understanding of specific job demands, resources, and health outcomes among virtual team members to improve working conditions and promote employee health. Given the challenge of operationalising virtuality, this study took a chance to provide a scale considering both, key elements of the definition of virtual teams as well as contemporary ways of team communication. Considering the reliability of this scale in this study, including the usage of digital media did not contribute to a better measurement of virtuality in this study in terms of reliability. The challenge of developing a measurement assessing virtuality as it is currently implemented seems to last for future research. Self-reports may have also affected objectivity in this study but were considered the most feasible way to obtain data. Moreover, we did not include a control group of “traditional teams” to compare the results of virtual teams to. Since the sample is relatively small and does not represent virtual teamwork in general, the external validity of the results is limited. Additionally, the dearth of research addressing health in virtual teamwork provides a very limited base of literature for a discussion of our results. Therefore, comparable working conditions, such as mobile or telework, needed to be adduced as well. The results, therefore, need to be interpreted cautiously. Lastly, the cross-sectional design and the small sample size of this pilot study can only provide a snapshot, but no opportunity for the interpretation of causal relationships. However, this pilot study served to generate first quantitative data on mental health in virtual teamwork. On this basis, a longitudinal study of a larger scale can be designed and conducted to gain a deeper knowledge of working conditions and health outcomes in virtual teamwork.

### Practical implications and future research

Nevertheless, our results suggest some practical implications: helping virtual team members to improve their working conditions might be beneficial for their sleep quality and thus their health and well-being. The results indicate that psychological detachment, as a personal recovery resource, can help virtual team members to improve their sleep quality. As a person-centred approach, mindfulness-based stress programmes and active leisure activities can have beneficial effects on recovery and support employees to detach from work [65, 66]. On an organisational level, increased job control and decreased workload may help to reduce the need for recovery [66]. Frequent, synchronous, and rich communication e.g., using video calls instead of emails, may help to reduce psychological distance between virtual team members [67, 68]. For a start, it is important to organise a kick-off event where virtual team members meet face-to-face [4]. Such a launch event can help to develop trust and confidentiality, facilitates communication and can prevent potential conflicts [4, 6]. It may be also helpful to establish binding rules of communication and documentation [11]. Individual support could be provided through feedback conversations and coaching [69].

For further research, it may be worthwhile to test especially boundarylessness in another quantitative study of a larger scale or longitudinal research design again since it did not appear as a significant job demand in this study. Future research may also want to examine other possible virtual job demands, such as technological stressors, coordinating efforts, conflict susceptibility, or information overload [6, 11, 70].

Operationalisation of virtuality remains a central challenge of future quantitative research, whether to rely on one single item [71] or complex indices [41]. The approach of including different media channels, frequency of usage, face-to-face meetings, spatial distance, distribution, and isolation, has not proven to be reliable in this study. However, the elimination of digital media usage from this scale resulted in sufficient reliability, providing a starting point for further research.

Another possible explanation may be obtained by examining self-regulation as an intervening variable that might reduce perceived stress [72]. Although it is important to investigate job demands and stressors, a focus on resources and coping strategies, such as resilience [73], will be necessary to deduce practical implications and develop concrete and target group-specific health promotion measures.

Although leadership in virtual teams has been addressed by researchers in the past already [74, 75], associations between leadership behaviour and employee health in virtual teamwork still need to be further examined [11]. First studies indicate that appreciative leadership was perceived as a resource among virtual team members [69].

## Conclusions

To our best knowledge, this pilot study was the first quantitative one to apply the JD-R Model to the context of virtual teamwork in Germany. The results provide new information on job demands, personal resources, and health outcomes among virtual team members. Considering methodological limitations, psychological detachment was found a valuable resource with regard to sleep quality for virtual team members. The differences among supervisors and employees shown in this coping strategy provide a basis for further research. The results highlight the relevance and urgency to further examine virtual teamwork-related demands, resources, and health outcomes to deduce appropriate prevention measures for virtual team members. Longitudinal research could provide further results that can contribute to a healthier way of working in virtual teams.

## Data Availability

The data analysed in this study are not publicly available due to German national data protection regulations.

## Abbreviations

*b*: Estimate value of regression coefficient in ordinal logistic regression analyses
CI: Confidence interval
COPSOQ: Copenhagen Psychosocial Questionnaire
Cronbach’s α: Measure for internal consistency (reliability)
ICT: Information and communication technologies
JD-R Model: Job Demands-Resources Model
*M*: Mean
*N*: Total number of cases (absolute number of participants)
*n*: Absolute number of cases in a subsample
*OR*: Odds ratio
*p*: Probability
*r*: Spearman correlation coefficient
*SD*: Standard deviation
*SE*: Standard error of the estimate
*t*: *t*-test value
